# Signatures of selection in tilapia revealed by whole genome resequencing

**DOI:** 10.1038/srep14168

**Published:** 2015-09-16

**Authors:** Jun Hong Xia, Zhiyi Bai, Zining Meng, Yong Zhang, Le Wang, Feng Liu, Wu Jing, Zi Yi Wan, Jiale Li, Haoran Lin, Gen Hua Yue

**Affiliations:** 1 Molecular Population Genetics and Breeding Group, Temasek Life Sciences Laboratory, 1 Research Link, National University of Singapore, 117604 Singapore; 2 State Key Laboratory of Biocontrol, Institute of Aquatic Economic Animals and Guangdong Provincial Key Laboratory for Aquatic Economic Animals, College of Life Sciences, Sun Yat-Sen University, Guangzhou 510275, PR China; 3 Key Laboratory of Exploration and Utilization of Aquatic Genetic Resources, Shanghai Ocean University, Ministry of Education, Shanghai 201306, China; 4 Key Laboratory of Freshwater Fisheries and Germplasm Resources Utilization, Ministry of Agriculture, Freshwater Fisheries Research Center, Chinese Academy of Fishery Sciences, Wuxi 214081, China; 5 Department of Biological Sciences, National University of Singapore, Singapore 117543, Singapore; 6 School of Biological Sciences Nanyang Technological University, 60 Nanyang Drive, Singapore, 637551, Singapore

## Abstract

Natural selection and selective breeding for genetic improvement have left detectable signatures within the genome of a species. Identification of selection signatures is important in evolutionary biology and for detecting genes that facilitate to accelerate genetic improvement. However, selection signatures, including artificial selection and natural selection, have only been identified at the whole genome level in several genetically improved fish species. Tilapia is one of the most important genetically improved fish species in the world. Using next-generation sequencing, we sequenced the genomes of 47 tilapia individuals. We identified a total of 1.43 million high-quality SNPs and found that the LD block sizes ranged from 10–100 kb in tilapia. We detected over a hundred putative selective sweep regions in each line of tilapia. Most selection signatures were located in non-coding regions of the tilapia genome. The Wnt signaling, gonadotropin-releasing hormone receptor and integrin signaling pathways were under positive selection in all improved tilapia lines. Our study provides a genome-wide map of genetic variation and selection footprints in tilapia, which could be important for genetic studies and accelerating genetic improvement of tilapia.

Genetic selection in which the best individuals are selected as parents of the next generation is the principal tool to improve crops and livestock^1^. The heritable variation, which is the basis for genetic improvement in agriculture, reflects the potential of a population to respond to selection^2^. Adaptation in response to selection on polygenic phenotypes may occur via subtle allele frequency shifts at many loci^3^. The search for genomic variations and selection signatures across the entire genome can bring new insights into genes that contribute most positively to the agronomic phenotypes of animals. A number of statistical methods for identifying selection signatures have been developed, such as Fay and Wu ’s H Test^4^, allele frequency differences^5^,^6^,^7^, EHH^8^, iHS (Integrated Haplotype Score)^9^ and Rsb^10^. With the advance of cost-effective and high throughput genotyping/sequencing methods, a number of genome-wide scans for selection signatures have already identified hundreds of regions targeted by recent positive selection in humans^8^,^11^ and livestock, such as cattle^12^, chicken^13^ and pig^14^. However, only a few studies on selection signatures have been conducted in aquacultured species, e.g., Channel catfish^15^, carp^16^, Atlantic salmon^17^, even though fish is one of the most important protein sources for humans.

Tilapia is the common name of a group of cichlid fishes native to the Middle East and Africa, and includes over 70 species^18^. In order to improve tilapia seed quality and diversify its production, a few tilapia breeding programs have been conducted since the 1980s, which have led to the current diversity in commercial tilapia lines. The GIFT (Genetically Improved Farmed Tilapia) project, which started in 1987, is the most successful tilapia breeding program in the world^18^. In order to establish a wide genetic base, a total of eight African and Asian tilapia founder populations were used in the GIFT genetic improvement program^19^. The GIFT strains have been introduced to more than 10 countries, such as China, Thailand, Malaysia and Indonesia. Tilapia has currently become the second most important fish in aquaculture after carps. The worldwide production of tilapia exceeded 3.5 million tons in 2010. Currently, Nile tilapia (*O. niloticus*), Blue tilapia (*O. aureus*), Mozambique tilapia (*O. mossambicus*) and various hybrids including Red tilapia are the tilapia species/strains commonly produced across the world^18^. Given its great economic importance, tilapia has also attracted increasing attention in the scientific field. So far, a large number of microsatellites for *O. aureus, O. niloticus*^20^,^21^ and *O. mossambicus* x *O. spp*^22^, as well as over thousands of single-nucleotide polymorphisms (SNPs) of Nile tilapia from next generation DNA sequencing data^23^,^24^ have been reported. Gene-associated SNPs derived from ESTs^25^ and RNA-seq data^26^ have also been discovered. Several linkage maps for Nile and Mozambique tilapia have been constructed^21^,^22^. Quantitative trait loci (QTL) for important traits including sex^22^,^23^,^27^,^28^ and growth traits^27^,^29^ have been identified in tilapia. Recently, the genome sequences of Nile tilapia and four other cichlid species were published^30^. The draft genome sequence of tilapia offers a reference genome for identifying genome-wide genetic variation that contributes to phenotypic diversity among/within tilapia lines and for detecting genome response to artificial selection in tilapia.

Adaptive differences can also be identified in genes subject to positive Darwinian selection^31^. Genome variations of aquacultured fish species, which are subject to artificial selection, are of particular interest as they may contribute positively to economic traits, such as growth and disease resistance. However, in genetically improved aquaculture species, the identity of the genome-wide genetic variation and how the genomes have been shaped by selective breeding remain unclear. In order to address these questions, we conducted a genome-wide scan for variation and signatures of recent selection in tilapia by resequencing the genomes of 47 tilapia samples originating from five populations found in South Africa, China and Singapore, representing the major genetically improved tilapia lines in Asia. This first selective sweep analysis identified a large number of SNPs, some interesting candidate genes and pathways related to economically important traits affected by selection, which could be important for genetic studies and accelerating genetic improvement of tilapia.

## Results and Discussion

### Sequencing, identification and annotation of genome-wide SNPs in tilapia

We selected 47 samples collected from South Africa, Singapore and China to represent the wild tilapia, and the major genetically improved lines in Asia. These samples included 23 GIFT tilapia, eight Nile-c tilapia, 12 Mozambique tilapia (8 Mzb-F0 and 4 Mzb-F2) and four Red tilapia (see details in Supplementary Table S1). Among these 47 fishes, eight Mozambique tilapia in population Mzb-F0, which were imported from a wild population in South Africa, were regarded as unselected tilapia. All others were from improved lines. The 23 GIFT tilapia originated from the GIFT population, which is a collection of selected GIFT tilapia strains (Supplementary Table S1). The 47 tilapia DNA samples were sent to Beijing Genome Institute (BGI) for 101 bp paired-end sequencing using Illumina Hiseq 2500. We obtained 300 Gb of raw sequencing data. After quality check and filtering, 254 Gb of high quality reads were used in analyzing variation and selection signatures. This high quality data covered 5.4-folds of the tilapia genome for each sample.

SNPs are valuable tools for analyzing economically important traits and other genetic studies. To identify the genome-wide SNPs in the tilapia genome, we first mapped our high quality reads to the reference genome (~665 Mb) of Nile tilapia^30^, which included 22 chromosomes and UNK1 supercontig. We found that the mapping rate in different individuals varied from 73.7% to 76.2%, which is slightly lower than the data reported in previous studies, such as in rice^32^. Most of the unmapped reads might have resulted from gaps in the current tilapia genome assembly, individual-specific or diverged sequences, or from sequencing errors. We identified SNPs for each individual. Using a strict Samtools-based SNP calling pipeline, 1.4 million putative SNPs were identified from the genomes of the 47 samples (Table 1). Detailed information about the SNPs is available upon request. Among the 22 tilapia chromosomes, the highest SNP number (105,510) was found on chromosome 7 whereas the lowest number (37,405) occurred on chromosome 10. The number of SNPs that were detected was positively correlated with the length of the chromosome (R = 0.99). We found that on average, there occurred one SNP in every 463 nucleotide base pairs along the tilapia genome (Table 1). This ratio is lower than that (200 bp/SNP) along the chicken genome^13^.

Validation of SNPs identified by whole genome resequencing was conducted by sequencing 96 randomly selected SNPs in 6 of the 47 samples with Sanger sequencing (Supplementary Table S2). The overall sequence concordance was 98.8%. Our data is comparable to the data in cattle (96.2%)^33^ and in the silkworm (96.7%)^34^. The high level of sequence concordance between NGS and Sanger sequencing indicates that the SNP calls in the NGS data are highly reliable. To our best knowledge, this study represents the largest high-quality SNP data set obtained in tilapia. This SNP data may be used to identify economically important genes and QTL, and to conduct GWAS for important traits to identify DNA markers to facilitate the genetic improvement of tilapia.

**Figure 1:**
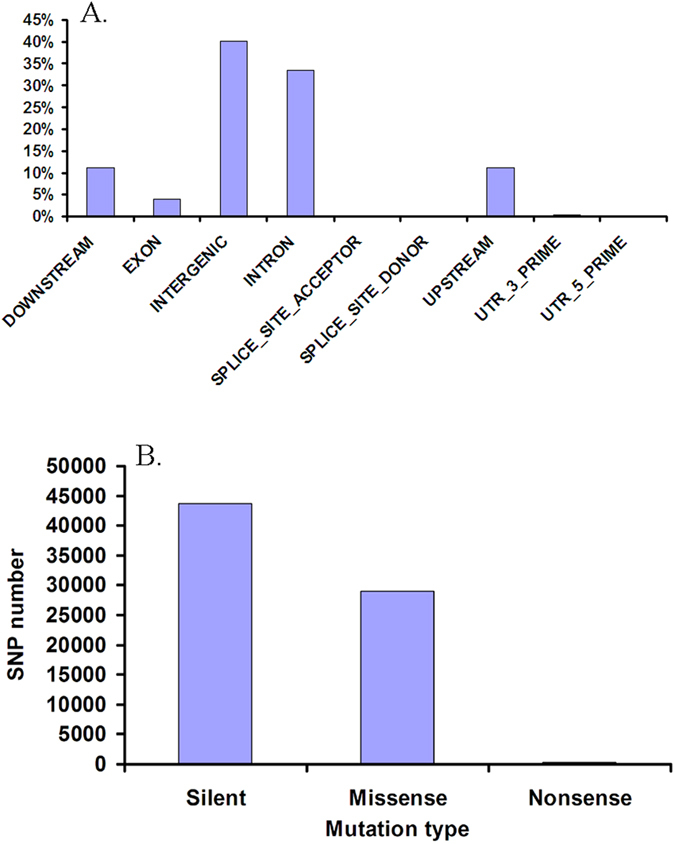
Percentage of SNPs in different locations in the tilapia genome and number of SNPs in each category of mutations in exons. (**A**) Percentage of SNPs in different locations in the tilapia genome; (**B**) Number of SNPs in each category of mutations in exons.

The distributions of SNPs in the tilapia genome were calculated using the toolbox SnpEff^35^ (Fig. 1A). The intergenic regions contained 40% of the SNPs, while the intron regions consisted of 33% of the SNPs, and only 4% of SNPs fell within exon regions. Transition to transversion ratio in SNPs was estimated to be 2.61, which is slightly higher than that (2.1) in humans^36^. Of the SNPs in the exon region, 60% were silent mutations and 40% were missense mutations (Fig.1B). The missense to silent ratio was 0.66, which is smaller than that in humans where the ratio of missense to silent mutations is close to 1^37^. The exonic variation identified in this study will provide valuable information for studies on functions of genes in tilapia.

### Genetic diversity and population structure among tilapia populations

**Figure 2:**
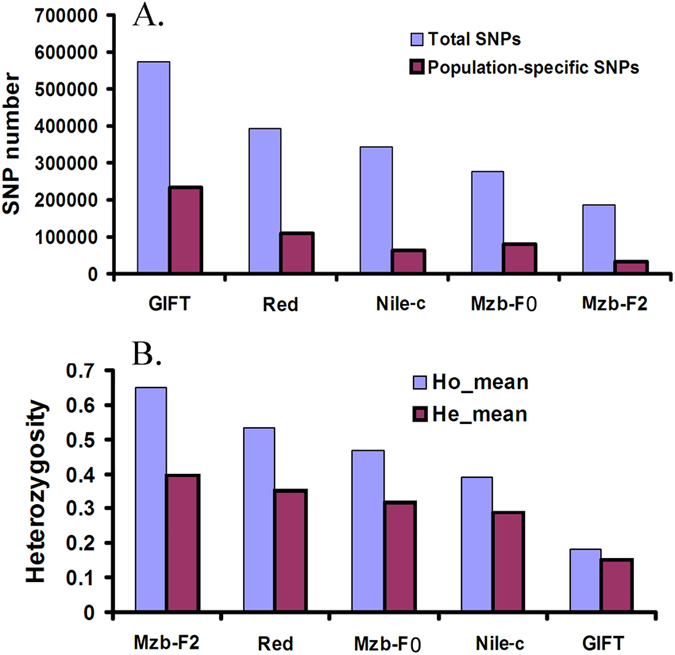
Number and heterozygosities of SNPs identified in five populations of tilapia. (**A**) Number of SNPs identified in five populations of tilapia; (**B**) Heterozygosities of SNPs in five populations of tilapia.

We evaluated the genetic diversity among populations based on the SNP data by calculating SNP numbers, observed heterozygosity (*Ho*) and expected heterozygosity (*He*). The total SNP number and population-specific SNP number are presented in Fig. 2A. We found that the highest *Ho* and *He* were in the Mzb-F2 population while the lowest *Ho* and *He* were seen in the GIFT population (Fig. 2B). The lowest *Ho* was identified in the GIFT tilapia, suggesting that world-wide cultured GIFT strains may have lost large amounts of genetic diversity due to inbreeding or founder effect. The GIFT tilapia having the highest SNP number was expected since the population was a mixture of different small stocks collected from several countries^18^. We also found that the SNP number in the Mzb-F2 population was lower than that in the Mzb-F0 population, which might have resulted from a stronger reduction in effective population size in the second generation of intensive selection, or smaller sample size used in the study. On the other hand, we found the highest observed heterozygosity in the Mzb-F2. However, these results are unexpected and not compatible with a theory in which the heterozygosity is expected to decline by a factor of 1/2Ne (Ne, effective population size) in an ideal population^38^. These might suggest that the sample numbers in these populations seem too small to predict the heterozygosity of population.

**Figure 3:**
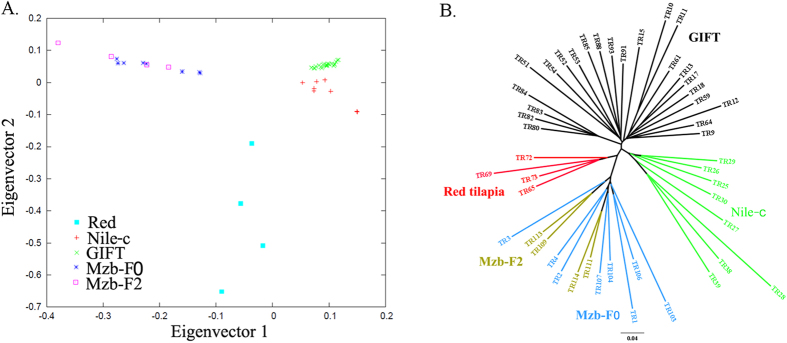
Population substructure in tilapia revealed by principal component analysis and phylogenetic tree analysis. (**A**) Principal component analysis. The five populations are shown in different colors and symbols; (**B**) Phylogenetic tree analysis. The five populations and clades are shown in different colors. The population ID and sample ID are presented.

To examine genetic population structure and relationships among the major groups of tilapia samples, we conducted a principle component analysis (PCA) and constructed a Neighbor-Joining (NJ) tree, based on the high-quality SNPs data. Using the first and second eigenvectors, the PCA clearly divided the 47 samples into three groups (Red tilapia, Mozambique tilapia and Nile tilapia) (Fig. 3A). All the Mzb-F0 and Mzb-F2 individuals were very closely related, showing apparent differentiation from Nile-c tilapia and Red tilapia, which is consistent with our previous work^26^. The GIFT and Nile-c populations were closely related but clearly separated from one another, suggesting relatively little subsequent gene flow between the two tilapia lines. This genetic difference could be because these two lines have been selectively bred to adapt to different environmental conditions. PCA showed a more dispersed population substructure in the Red tilapia strain, which may be due to the cross between Mozambique and Nile tilapia during the breeding of the red tilapia^26^.

A NJ tree was used to cluster samples based on average genetic distances (Fig. 3B). The phylogenetic analysis showed a similar population structure as the one generated with the PCA. The NJ tree contained four major groups, corresponding to Red tilapia, Mozambique tilapia, GIFT and Nile-c tilapia. As expected, the phylogenetic analysis incorporated the Mzb-F0 and Mzb-F2 into one clade. The GIFT strains were separate from Nile-c tilapia, indicating independent domestication/breeding, which is in agreement with the actual breeding process of the two lines^18^. The four Red samples were closely related to Mozambique tilapia and were clustered into one clade, suggesting a different domestication/breeding event from Nile and Mzb populations. These results are consistent with our previous study based on EST-originated SNP markers^26^ where significant population structure in tilapia was detected through phylogenic and population structure analysis. Taken together, our data suggest that there are significant population structures among the tilapia populations.

### Analysis of linkage disequilibrium (LD) decay in tilapia

**Figure 4:**
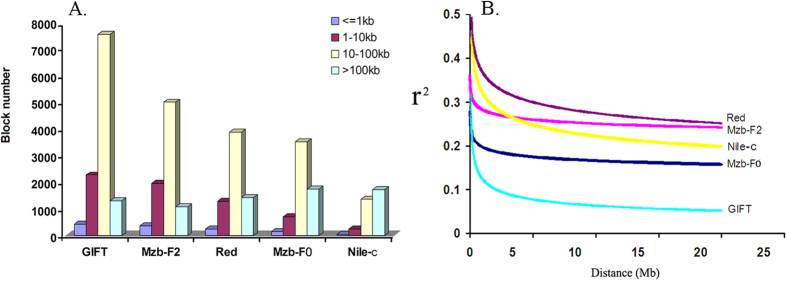
Number of different size LD blocks (A) and LD decay detected in the five populations of tilapia (B).

LD is useful for determining the number of markers that are needed for an association study. To estimate the LD patterns in the different tilapia groups, we calculated squared correlations of allele frequencies against genome distance (r^2^) between pairs of SNPs using Haploview^39^. We found slight differences among the populations at the LD block size >100 kb. However, at the block size of <100 kb, the highest LD block number was detected in GIFT strains and the lowest LD block number in Nile-c tilapia (Fig. 4A). Except the Nile-c tilapia population, most of the LD block sizes in the tilapia populations ranged from 10–100 kb. The plot of LD decay pattern against the genome distances is shown in Fig. 4B. Our study revealed that the LD level decreased faster in Nile-c and GIFT compared to other populations. In many previous studies, domesticated strains often showed longer LD than wild populations^40^. It is interesting that the GIFT population had the lowest LD level while the Red population had the highest LD level. This may suggest that the LD level in a population is more complex, and may be associated with the genetic background and breeding history of the strains. During the selection of the GIFT strain, eight founder populations form both wild and farmed populations were used^19^. The samples used in this study consisted of small groups collected from Shanghai and Guangzhou of China and Singapore, which have been selected for growth for over 15 generations since 1987. However, the detailed cross-breeding history of the GIFT strains used in the study is not known. To address the LD issues clearly, we have to characterize more samples in the future. Nevertheless, the information about LD patterns in each tilapia group would be useful in selecting SNPs for genome-wide association studies to identify markers associated with economically important traits on the whole genome level.

### Signatures of positive selection revealed by iHS statistics

Population genomics has offered a new paradigm for detecting signatures of selection. To detect selective sweeps driven by artificial selection and natural selection in cultured tilapia, we used the iHS statistic^9^, which detects the evidence of recent positive selection at a locus by searching for genomic regions with excess homozygosity. We conducted a sweep analysis in four tilapia breeding populations. By using the iHS test, we found a total of 1120 extreme iHS values exceeding the empirical threshold level of 2 across the four populations. There were 163, 362, 243 and 352 outliers detected in the Mzb-F2, Nile-c, GIFT and Red populations, respectively (Supplementary Tables S3, S4, S5 and S6). We annotated the SNPs with the software SnpEff^35^ by using the tilapia genome annotation information to identify candidate genes that underwent sweeps. Annotation of the regions harboring clustered iHS signals revealed 115, 243, 151 and 219 candidate genes in the Mzb-F2, Nile-c, GIFT and Red populations, respectively. Some of these candidate genes (Supplementary Tables S3, S4, S5 and S6), which were under artificial or natural selection, are novel and have not been functionally annotated yet.

**Figure 5:**
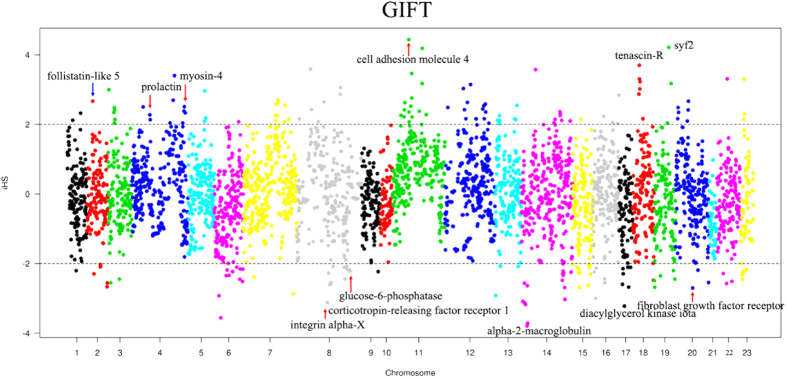
The genome-wide sweep analysis based on iHS statistics for the GIFT tilapia population. The iHS value at a locus on each chromosome are shown in the figure. Some genes with significant SNPs when using an iHS threshold of ±2 are shown. The LG8_24, LG16_21 and Unk1 scaffold are shown as Chromosome 8, 16 and 21 for clarity.

The GIFT population has experienced intense artificial selection. The GIFT strains, when compared to the wild Nile tilapia populations, have gained some significant advantages in aquaculture, such as resistance to handling conditions and fast growth. In the sequencing data of the GIFT strains, a substantial number (135 out of 243 cases) of SNPs with extreme iHS values were found to be positioned in intergenic regions. Among them, 101 were located in introns, 22 in upstream and 26 in the downstream regions (Supplementary Table S5). Nonsense mutations, which are obvious candidates of functional significance, may have contributed to the rapid evolution in domestic animals^41^. However, in the GIFT tilapia, only one SNP was a non-synonymous mutation, which was located at 21 Mb on chromosome LG14 annotated as extracellular calcium-sensing receptor-like. A panel of interesting candidate genes containing SNPs with extreme iHS values responding to the artificial selection was identified, such as prolactin and myosin-4 in the GIFT population (Fig. 5). Previous studies showed significant associations of their homologous genes with economic traits in animals^42^,^43^, supporting the notion that these genes could be selected during breeding for genetic improvement. These candidate genes under selection identified in our study could provide useful information for rapidly identifying DNA markers associated with economically important traits in Tilapia.

In the other tilapia populations, most (more than 80%) SNPs under selection could be clustered into intergenic regions and introns. Only 3, 6 and 7 non-synonymous SNPs were detected in the Red, Mzb-F2 and Nile-c populations, respectively (Supplementary Tables S3, S4 and S6). We found non-synonymous SNPs in genes OR11A1, EPS15L1 and EFNA2 in the red population; OR11A1, hypothetical protein LOC100700781, CDAN1, MR1, hypothetical protein LOC100690504 and zwilch homolog in the Mzb-F2 population; and IL13RA2, WDFY3, GPRC6A, PDK1, Slc9a3, Anpep and HPS5 in the Nile-c population. A previous study showed that WDFY3 was associated with individual piglet birth weight in a genome-wide association study^44^, and that knockout of the GPRC6A receptor increased susceptibility to diet-induced obesity in mice^45^. PDK1 is essential for mouse embryonic development, and regulates cell size^46^. The presence of low numbers of nonsense and missense mutations suggests that gene inactivation has not played a prominent role during domestication/breeding. This is consistent with the findings in pig^47^ and chicken^13^. Further studies on the functions of the genes and regulatory regions under selection may facilitate the comprehensive understanding of the molecular mechanisms underlying phenotypic variations during domestication and breeding in aquaculture species.

### Gene ontology and pathway analysis for candidate genes under selection

**Figure 6:**
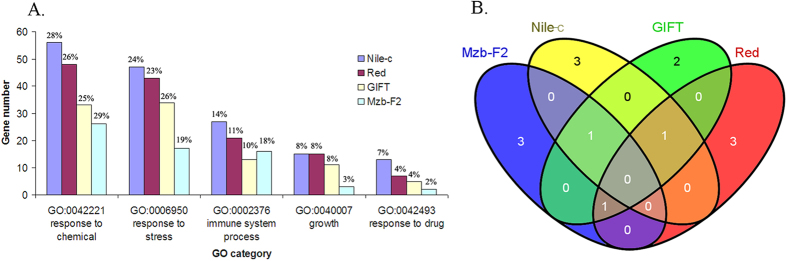
Number of genes in different ontology categories (A) and number of overlapping pathways containing genes under selection among four genetically improved tilapia populations (B).

Selection signatures under artificial selection can be detected by first scanning the genome regions, and then identifying the corresponding selected phenotype and adaptive pathways^12^. To further explore the functions of the selective sweep gene candidates in detail, we annotated them by using the gene ontology database and pantherdb pathway database (http://www.pantherdb.org/geneListAnalysis.do). The candidate genes from each population were mapped into five gene ontology categories (response to chemical, response to stress, immune system process, growth, response to drug) that are thought to be important to tilapia performance (Fig. 6A). We found 25–29% of the genes in response to chemical, 19–23% in response to stress and 10–18% that play roles in the immune system, suggesting that the genes in these categories may be frequently under adaptive selection. We also found that 3–8% of genes identified were related to growth (Fig. 6A), which is a trait extensively selected in breeding programs of aquaculture species^48^.

We mapped these gene candidates to known pathways. The top five pathways that were identified for the GIFT population were the EGF receptor signaling pathway (P00018), FGF signaling pathway (P00021), Gonadotropin releasing hormone receptor pathway (P06664), integrin signaling pathway (P00034) and Wnt signaling pathway (P00057) (Table S7). These pathways have been found to play important roles in animal growth, development and diseases^49^. To identify pathways affected by artificial or natural selection among populations, we examined pathways, which overlapped among the four populations (Fig. 6B.). We found that three pathways were the same in three populations, namely the Wnt signaling pathways, gonadotropin-releasing hormone (GnRH) receptor pathway and integrin signaling pathway. A previous study showed that the Wnt signaling pathways played a role in carcinogenesis and in embryonic development^50^. The GnRH receptor is a key regulator of the reproductive system and fecundity^51^. Integrins are cell surface receptors, which respond to the growth factor signaling pathways^52^. In laying chicken, the integrin signaling pathway was believed to be perturbed during its domestication and/or due to selection for egg production^53^. Taken all together, our study suggests that the genes in these three pathways may be most likely influenced by artificial selection for growth in fish. The line-specific selective sweep genes and pathways in each selected line could be independently selected in each line, which may be important to the development of morphological and physiological differences to adapt to different environmental conditions for different lines.

In summary, we present the first comprehensive study for localizing signatures of selection in tilapia based on full re-sequencing data. A total of 1.4 million high-quality SNPs were identified by resequencing 47 tilapia samples, representing major cultured tilapia strains in Asia. Population structure and phylogenetic analysis using genome-wide SNPs clearly separated the GIFT, Red and Mozambique tilapia into three different groups. The selective sweep analysis revealed some interesting candidate genes and pathways under selection. The pathways affected by artificial or natural selection include the Wnt signaling, GnRH receptor and integrin signaling pathways. Most selection signatures were located in non-coding regions of the genome of tilapia. The data of our study could be important for speeding up genetic improvement of the tilapia populations and studying the mechanisms underlying phenotypic variations.

## Materials and Methods

### Sample collection, ethnic statement, DNA sequencing, alignment and SNP calling

Tilapia samples were collected from South Africa, China and Singapore. The detailed information about these samples is listed in Supplementary Table S1. All handling of fishes was conducted in accordance with the guidelines on the care and use of animals for scientific purposes set up by the Institutional Animal Care and Use Committee (IACUC) of the Temasek Life Sciences Laboratory (TLL), Singapore. The IACUC of TLL has specially approved this study within the project “Breeding of Tilapia” (approval number TLL (F)-12-004).

Genomic DNA was extracted from each fish using a DNeasy Blood & Tissue Kit (Qiagen, Valencia, CA, USA) according to the manufacturer’s instructions. DNA samples were sent for sequencing (101-bp pair-end reads) using Illumina HiSeq 2500 at Bejing Genome Institute (BGI, Shenzhen, China). Paired-end data were then processed to filter out the low quality reads and adaptors using the software NGS QC Tool kit^54^ (./perl IlluQC.pl –pe file.fq 2 A –p 10 –o outputfolder). The Nile tilapia reference genome (Broad Institute Tilapia anchored genome assembly v1.1) and corresponding genome annotation GFF3 file were downloaded from the website (http://cichlid.umd.edu/cgi-bin/gb2/gbrowse/Tilapia_broad_anchored_v1/?source=Tilapia_broad_anchored_v1). The software packages Bowtie 2^55^ and SAMtools^56^ were applied for aligning sequencing reads to the reference genome of tilapia. Calling SNPs from the data was performed with SAMtools/BCFtools (./samtools mpileup -Dugf reference.fasta sample.bam |./bcftools/bcftools view -bvcg - > raw.bcf; ./bcftools/bcftools view raw.bcf | ./bcftools/vcfutils.pl varFilter -D 100 | awk ‘{if (/^#/ || $6>30) print }’ > flt.vcf) and then filtered with the stringent parameters setting of: mapping Qual ≥ 46, base quality ≥ 20, qual ≥ 30, gq ≥ 20, mq ≥ 30, fq ≥ 20, 7 < ADP < 31 using internal linux commands. All indels were removed. Detailed information about the SNPs is available upon request. The raw sequence data are available under the DNA Data Bank of Japan (DDBJ) Sequence Read Archive database (BioProject Accession: PRJDB1657).

### Validation of SNPs by using Sanger sequencing

To validate the SNPs that were identified from the NGS data, 96 SNP sites from the NGS dataset were randomly selected for validation of SNPs using Sanger sequencing. Primers (Supplementary Table S2) were designed in the region flanking the SNP in each sequence using PrimerSelect (DNAstar, CA, USA). PCR reactions for sequencing were then carried out using 6 individuals from the 47 samples used for NGS as DNA templates. PCR products were sequenced by using Sanger sequencing. The genotypic data for each individual were manually called. Comparison of the Sanger genotypic data to the NGS dataset was conducted.

### Analysis of population structure, genetic diversity and linkage disequilibrium (LD)

Principle component analysis (PCA) was conducted with the software EIGENSOFT (Ver. 5.0.1)^57^. We used the program SNPhylo^58^ to estimate the population origin and admixture of the sequenced fishes. With the filter parameters (M 0.3, m 0.05, -p 15, -l 0.15), phylogenetic analysis was conducted using SNPs. A Neighbor-Joining (NJ) tree was constructed by using the software Clustal X^59^ with a bootstrap of 1000. Heterozygosity for each locus, LD and haplotype blocks were analyzed using Haploview (Ver.4.2)^39^ with the parameters: max distance 1000 kb, -dprime –min MAF 0.001, HW cutoff 0.001.

### Identification of selection signatures and gene ontology (GO), and analysis of pathways

The footprints of recent or ongoing selection were detected by the tool Rehh using EHH-related statistics with default setting^60^. Annotation and effect prediction for SNPs with extreme iHS scores were performed using the toolbox SnpEff^35^ (java -Xmx16g -jar snpEff.jar eff -v tilapia_genome_database.1 -i txt SNP_with_extreme_iHS.txt). During annotation analysis, the SnpEff database were first constructed using the Nile tilapia reference genome (Broad Institute Tilapia anchored genome assembly v1.1) and corresponding genome annotation GFF3 file. The SNP sites with extreme iHS scores were then annotated with the toolbox SnpEff. GO Slimmer (http://tools.bioso.org/cgi-bin/amigo/slimmer) was used to examine if the selected genes were enriched for specific functional annotation clusters. Pathway analysis was conducted using the online tool Panther Classification System (http://www.pantherdb.org/geneListAnalysis.do) with default parameters.

## Additional Information

**How to cite this article**: Hong Xia, J. *et al.* Signatures of selection in tilapia revealed by whole genome resequencing. *Sci. Rep.* **5**, 14168; doi: 10.1038/srep14168 (2015).

## Supplementary Material

Supplementary Table S1

Supplementary Table S2

Supplementary Table S3

Supplementary Table S4

Supplementary Table S5

Supplementary Table S6

Supplementary Table S7

## Figures and Tables

**Table 1:** The length of each chromosome, number of SNPs and bp/SNP in each chromosome in the tilapia genome.

Chromosome	Length (bp)	No. of SNPs	bp/SNP
LG1	31,194,787	66,667	467
LG2	25,048,291	54,674	458
LG3	19,325,363	45,994	420
LG4	28,679,955	62,090	461
LG5	37,389,089	76,982	485
LG6	36,725,243	78,467	468
LG7	51,042,256	105,510	483
LG8_24	29,447,820	63,620	462
LG9	20,956,653	51,939	403
LG10	17,092,887	37,405	456
LG11	33,447,472	65,518	510
LG12	34,679,706	75,111	461
LG13	32,787,261	70,323	466
LG14	34,191,023	72,524	471
LG15	26,684,556	64,112	416
LG16_21	34,890,008	75,132	464
LG17	31,749,960	67,295	471
LG18	26,198,306	54,807	478
LG19	27,159,252	62,063	437
LG20	31,470,686	65,221	482
LG22	26,410,405	59,341	445
LG23	20,779,993	44,394	468
UNK1	7,888,616	16,904	466
**Total**	**665,239,588**	**1,436,093**	**463**
