# Supplementary material for: Signatures of selection in tilapia revealed by whole genome resequencing
Source: Sci Rep. 2015 Sep 16;5:14168. doi: 10.1038/srep14168 (PMC4570987; doi:10.1038/srep14168)
Supplement: Supplementary Table S1 [file srep14168-s1.pdf]

Supplementary information

Signatures of selection in tilapia revealed by whole genome resequencing

Jun Hong Xia [1,2,*], Zhiyi Bai [3], Zining Meng [2], Yong Zhang [2], Le Wang [1], Feng Liu [1], Wu Jing [4], Zi Yi Wan [1], Jiale Li [3,], Haoran Lin [2] & Gen Hua Yue [1, 5, 6, *]

1. Molecular Population Genetics and Breeding Group, Temasek Life Sciences Laboratory, 1 Research Link, National University of Singapore, 117604 Singapore.
2. State Key Laboratory of Biocontrol, Institute of Aquatic Economic Animals and Guangdong Provincial Key Laboratory for Aquatic Economic Animals, College of Life Sciences, Sun Yat-Sen University, Guangzhou 510275, PR China.
3. Key Laboratory of Exploration and Utilization of Aquatic Genetic Resources, Shanghai Ocean University, Ministry of Education, Shanghai 201306, China.
4. Key Laboratory of Freshwater Fisheries and Germplasm Resources Utilization, Ministry of Agriculture, Freshwater Fisheries Research Center, Chinese Academy of Fishery Sciences, Wuxi 214081, China.
5. Department of Biological Sciences, National University of Singapore, Singapore 117543, Singapore.
6. School of Biological Sciences Nanyang Technological University 60 Nanyang Drive Singapore 637551, Singapore.

*Correspondence to Dr Jun Hong Xia, email: xiajunh3@mail.sysu.edu.cn

*Correspondence to Dr. Gen Hua Yue, email: genhua@tll.org.sg

Supplementary Table S1 Detailed information about the 47 tilapia samples

Population name	Species	Source	Description	No. of samples
Mzb-F0	Mozambique tilapia	South Africa	A wild population including over 1000 Mozambique tilapia individuals that were introduced into Singapore from South Africa in 2011. This population has been used by us as a founder population for selection for growth traits since 2011.	8
Mzb-F2	Mozambique tilapia	Singapore	A second generation of Mozambique tilapia selected for growth traits from the wild population imported from South Africa in 2011.	4
Nile-c	Nile tilapia	Shanghai	A selected line of Nile tilapia originally introduced from Egypt into China in the 1980s. The line has been selected for growth traits for over 15 generations.	8
GIFT	Nile tilapia	Shanghai, Guangzhou and Singapore	A collection of GIFT strains, which have been selected for growth for over 15 generations since 1987.	23
Red	Red tilapia	Guangzhou of China	A selected population imported into China from Taiwan in 1983. The population has been selected over 10 generations in China.	4
